# Supplementary material for: ChatGPT as a Tool for Biostatisticians: A Tutorial on Applications, Opportunities, and Limitations
Source: Stat Med. 2025 Oct 23;44(23-24):e70263. doi: 10.1002/sim.70263 (PMC12548020; doi:10.1002/sim.70263)
Supplement: Supplementary file 1 — Data S1. Supporting Information. [file SIM-44-0-s001.zip › Supplementary Materials Additional.zip!/3.4_Sample_Sizes_Planning_for_Survival_Outcomes/3.4_Sample_Sizes_Newer_Versions/02_chat_sample_size_o4mini.pdf]

02_chat_sample_size_o4mini

Please clear memory

All set—I've cleared our prior conversational context. How can I assist you now?

You take the role of an expert biostatistician in this chat.

Understood—I'll be your expert biostatistician. How can I assist you today?

I would like to compare a new therapy with the standard therapy regarding survival in advanced pancreatic carcinoma. Please perform a sample size calculation and incorporate the following parameters:
o Primary outcome: Overall survival.
o Median survival time in the standard therapy group: 12 months.
o Median survival time in the new therapy group: 18 months.
o Power = 80% (1 - β = 0.8)
o a two-sided significance level of α = 0.05.
o The allocation ratio should be 1:1 (equal group sizes).
o Patients will be followed up for 2 years.
o We expect a dropout rate of 15%.
Use the data analysis mode

Sample Size Calculation Summary

	Parameter	Value	
1	Events required	191.0	
2	Avg. event probability at 24 months	0.677	
3	Total N before dropout	283.0	
4	Total N adjusted for 15% dropout	333.0	
[illegible]	N per group	[illegible]	

Based on an exponential-survival assumption and a two-sided log-rank test (α = 0.05, 80% power), comparing medians of 12 vs. 18 months over a 24-month follow-up yields:

- **Events required**: ~191
- **Average event probability by 24 months**: ~0.677
- **Total sample before dropout**: ~283 patients
- **Total sample after 15% dropout adjustment**: ~333 patients
- **Per-arm sample size (1:1 allocation)**: **167 patients**

Conclusion: Recruit **167 subjects per arm** (334 total) to achieve 80% power, accounting for 15% loss to follow-up. Let me know if you'd like to explore alternative assumptions or designs!
